# Association between ERCC1 and TS mRNA levels and disease free survival in colorectal cancer patients receiving oxaliplatin and fluorouracil (5-FU) adjuvant chemotherapy

**DOI:** 10.1186/1471-230X-14-154

**Published:** 2014-08-29

**Authors:** Sheng Li, Liangjun Zhu, Li Yao, Lei Xia, Liangxi Pan

**Affiliations:** Oncology, Jiangsu Tumor Hospital, NO.42 bai zi ting, Nanjing, 210000 China; Department of Hematology, First Hospital Affiliated to Suzhou University, Suzhou, 215000 China; Department of Pathology, Jiangsu Tumor Hospital, Nanjing, 210000 China

**Keywords:** Colorectal cancer, ERCC1, TS, Real-time PCR, Adjuvant chemotherapy

## Abstract

**Background:**

Aim was to explore the association of *ERCC1* and *TS* mRNA levels with the disease free survival (DFS) in Chinese colorectal cancer (CRC) patients receiving oxaliplatin and 5-FU based adjuvant chemotherapy.

**Methods:**

Total 112 Chinese stage II-III CRC patients were respectively treated by four different chemotherapy regimens after curative operation. The TS and ERCC1 mRNA levels in primary tumor were measured by real-time RT-PCR. Kaplan–Meier curves and log-rank tests were used for DFS analysis. The Cox proportional hazards model was used for prognostic analysis.

**Results:**

In univariate analysis, the hazard ratio (HR) for the mRNA expression levels of *TS* and *ERCC1* (logTS: HR = 0.820, 95% CI = 0.600 - 1.117, *P* = 0.210; logERCC1: HR = 1.054, 95% CI = 0.852 - 1.304, *P* = 0.638) indicated no significant association of DFS with the *TS* and *ERCC1* mRNA levels. In multivariate analyses, tumor stage (IIIc: reference, *P* = 0.083; IIb: HR = 0.240, 95% CI = 0.080 - 0.724, *P* = 0.011; IIc: HR < 0.0001, *P* = 0.977; IIIa: HR = 0.179, 95% CI = 0.012 - 2.593, *P* = 0.207) was confirmed to be the independent prognostic factor for DFS. Moreover, the Kaplan-Meier DFS curves showed that *TS* and *ERCC1* mRNA levels were not significantly associated with the DFS (*TS*: *P* = 0.264; *ERCC1*: *P* = 0.484).

**Conclusion:**

The mRNA expression of *ERCC1* and *TS* were not applicable to predict the DFS of Chinese stage II-III CRC patients receiving 5-FU and oxaliplatin based adjuvant chemotherapy.

## Background

Colorectal cancer (CRC) is the third most common cancer worldwide and has a high mortality rate [1]. About 608,000 deaths from CRC are estimated worldwide, accounting for 8% of all cancer deaths [2, 3]. Surgery is the most common treatment for CRC, yet prognosis remains poor [4]. As a result, considerable interest has concentrated on chemotherapy after surgery, such as oxaliplatin or 5-fluorouracil (5-FU)-based adjuvant chemotherapy [5, 6]. The 5-FU is an analogue of uracil with a fluorine atom at the C-5 position in place of hydrogen, which disrupts RNA synthesis and the action of thymidylate synthase by converting to several active metabolites: fluorodeoxyuridine monophosphate (FdUMP), fluorodeoxyuridine triphosphate (FdUTP) and fluorouridine triphosphate (FUTP) [7]. It was reported that 5-FU-based chemotherapy was a safe and effective treatment for elderly patients with advanced CRC [8]. Oxaliplatin is a platinum-based drug that has demonstrated antitumor activities in CRC both *in vitro* and *in vivo* [9]. It was reported that oxaliplatin based chemotherapy could significantly increase the progression-free survival in patients with metastatic CRC [10]. Moreover, the better efficacy and safety of oxaliplatin combined with 5-FU as first-line chemotherapy for elderly patients with metastatic CRC has been proved [11]. However, there is no predictive factor for efficacy of these treatments.

The *ERCC1* (encodes excision cross-complementing 1) gene codes for a nucleotide excision repair protein involved in the repair of radiation and chemotherapy-induced DNA damage [12]. It has been reported that the gene polymorphism of *ERCC1* at codon 118 was a predictive factor for the tumor response to oxaliplatin/5-FU combination chemotherapy in patients with advanced CRC [13]. Furthermore, thymidylate synthase (TS), as a target enzyme of 5-FU, is associated with response to 5-FU in human colorectal and gastric tumors [14, 15]. It was reported that *TS* genotyping could be of help in predicting toxicity to 5-FU-based chemotherapy in CRC patients [16]. However, little is known about the association between mRNA expression levels of *ERCC1* and *TS* and clinical outcomes of oxaliplatin and 5-FU based adjuvant chemotherapy in Chinese people with CRC.

In this study, we investigated the association of *ERCC1* and *TS* mRNA levels with the disease free survival (DFS) in Chinese CRC patients receiving oxaliplatin and 5-FU based adjuvant chemotherapy.

## Methods

### Patients

This study was carried out with the Institutional Ethics Committees approval and following the Chinese Medical Research Council guidelines. All participants gave their written informed consent prior to entering the study.

This is a prospective study. A total of 112 Chinese CRC patients who were treated at Jiangsu Tumor Hospital, China, from May 2005 to January 2010 were investigated in this study. Eligibility criterion was histological confirmation of stage II-III CRC after surgery according to the AJCC TNM classification [17].

### Chemotherapy treatment

All the patients were treated with chemotherapy after curative operation. Four types of chemotherapy regimens were used for the treatment of CRC patients: i) the first one was the standard FOLFOX-4 consisting of 2-hour intravenous infusion of oxaliplatin (85 mg/m^2^) on day 1, and 2-hour intravenous drip infusion of calcium folinate (200 mg/m^2^) on days 1–2, followed by intravenous injection of 5-FU (400 mg/m^2^) and continuous infusion of 5-FU (600 mg/m^2^) lasting 22 h on days 1–2, every 2 weeks; ii) the second one was the modified FOLFOX consisting of intravenous infusion of oxaliplatin (130 mg/m^2^) and 2-hour intravenous drip infusion of folinate calcium (200 mg/m^2^) on day 1, followed by intravenous injection of 5-FU (400 mg/m^2^) and continuous infusion of 5-FU (1000 mg/m^2^) over 24 h on days 1 to 3, every 3 weeks; iii) the third one was oral XELOX consisting of 2-hour intravenous infusion of oxaliplatin 130 mg/m^2^ on day 1 plus oral capecitabine 850 mg/m^2^ twice daily for 2 weeks in a 3-week cycle; iv) the fourth one was a conventional intravenous drip infusion including 2-hour intravenous infusion of oxaliplatin 130 mg/m^2^ on day 1 and continuous infusion of 5-FU (750 mg/m^2^) lasting 4 h on days 1 to 5, every 3 weeks. All the chemotherapy regimens were performed by a trained nurse. The selection of chemotherapy regimens for each patient was according to the recommendation of an experienced expert.

After treatment, the clinical outcomes were obtained by telephone follow-up or a return visit with the deadline of January 2014. DFS, which defined as the time from the end of chemotherapy to the first event of either recurrent disease or death, was calculated according to follow-up data.

### RNA extraction and real-time RT-PCR

RNA was extracted and purified from formalin-fixed paraffin-embedded (FFPE) tissue samples of surgically resected primary CRC using an RNeasy mini kit (Qiagen, Inc.) according to the manufacturer’s instructions [18]. The cDNA of *ERCC1* and *TS* was prepared by reverse transcription from RNA [19]. The ABI PRISM 7700 Sequence Detection System (Perkin-Elmer Applied Biosystems, Foster City, CA) was used to perform TaqMan probe-based real-time PCR reactions as previously described [20–22]. Relative levels of mRNA transcripts were calculated according to the comparative Ct method using *β-actin* as an endogenous control [23].

### Statistical analysis

The Cox proportional hazards model was used for univariate and multivariate analysis of prognostic factors. The variables included six continuous variables (age, duration of chemotherapy courses, interval between surgery and chemotherapy, cumulated dosage of oxaliplatin and mRNA expression levels of *ERCC1* and *TS*) and eight categorical variables (sex, primary tumor location, tumor stage, tumor differentiation, lymph node staging, nerve invasion, vascular invasion and chemotherapy regimens). The logarithms of the *TS* and *ERCC1* mRNA levels (logTS, logERCC1) were calculated for fitting normal distribution as the requirement of analysis. Dummy variables were considered for all the categorical variables. The chemotherapy regimens were used as a stratification variable in all the analyses. The backward stepwise method was used in the multivariate analysis base on the likelihood ratio statistics. Kaplan–Meier curves and log-rank tests were used for DFS analysis. Hazards ratios were used to calculate the relative risks of recurrence or death. All tests were two-sided, and *p* < 0.05 was considered as statistically significant. Analyses were performed using SAS version 9.1 (Institute, Cary, NC) and SPSS 19.0 (IBM, Armonk, New York). Power was calculated using the PS Power and Sample Size Calculation, version 3.0.43 (Vanderbilt University, Nashville, TN, USA).

## Results

### Characteristics of patients and follow-up results

Demographic details on the patients investigated in this study are shown in Table 1. A total of 112 Chinese patients (40 females and 72 males) aged from 32 to 75 years old (average, 52.75) were analyzed in this study. There were 61 rectum cancer patients and 38 colon cancer patients. All patients (stage IIa, 24; stage IIb, 1; stage IIIa, 3; stage IIIb, 53; stage IIIc, 31) underwent curative operation and then received 4 different chemotherapy regimens, respectively, including the standard FOLFOX-4 (20 cases), modified FOLFOX (15 cases), oral XELOX (19 cases) and conventional intravenous drip infusion (58 cases). The median follow-up duration was 36 months (ranged from 1.2 to 78 months). Relapse occurred in forty-four patients (39.3%) and eleven patients (9.8%) died of disease. The median DFS was 36 months (minimum: 3 months; maximum: more than 77 months) (Table 1).

**Table 1:** **Demographic and clinical parameters of patients** (*n* = 112)

Characteristics	Patients	
	No.	%
*Age* (years)		
Mean	52.72	
Range	32-75	
*Sex*		
Female	40	35.70%
Male	72	64.30%
*Lymph node staging*		
N0	25	22.32%
N1	49	43.75%
N2	38	33.93%
*Tumor stage*		
Stage IIa	24	21.40%
Stage IIb	1	0.90%
Stage IIIa	3	2.70%
Stage IIIb	53	47%
Stage IIIc	31	27.60%
*Primary tumor location*		
Rectum	61	54.46%
Colon	38	33.93%
*Vascular invasion*		
Positive	15	13.39%
Negative	97	86.61%
*Nerve invasion*		
Positive	24	21.43%
Negative	88	78.57%
*Chemotherapy regimen*		
Standard FOLFOX-4	20	17.80%
Modified FOLFOX	15	13.40%
Oral XELOX	19	17%
Conventional intravenous drip infusion	58	51.70%
*Interval of chemotherapy and surgery*		
Within 28 days	36	32.14%
More than 28 days	76	67.86%
*Duration of chemotherapy course*		
1-6 weeks	21	18.75%
7-12 weeks	25	22.32%
13-18 weeks	58	51.80%
19-24 weeks	8	7.14%
*Tumor differentiation*		
High	1	0.8%
High or medium	2	1.7%
Medium	64	57.1%
Medium or low	29	25.9%
Low	16	14.3%
*Follow-up*		
Median	36 months	
Range	1.2-78 months	
Relapse	44	39.3%
Death	11	9.8%
*Disease free survival*		
Median	36 months	
Range	3-77 months	

### The mRNA expression levels of TS and ERCC1

The median mRNA expression level of *TS*, relative to the housekeeping gene *β-actin,* was 2.86 × 10^-1^ (minimum expression, 2.7 × 10^-2^; maximum expression, 6.31). The median mRNA expression level of *ERCC1*, relative to the housekeeping gene *β-actin*, was 1.7 × 10^-3^ (minimum, 8.57 × 10^-5^; maximum, 6.7 × 10^-2^). In addition, when analyzed by sex and age, no significant association between the *TS* or *ERCC1* mRNA levels and these parameters was found (*P* > 0.05).

### Association of DFS with TS and ERCC1 mRNA levels

For the univariate analysis, the hazard ratio (HR) for the mRNA expression levels of *TS* and *ERCC1* (logTS: HR = 0.820, 95% CI = 0.600 - 1.117, *P* = 0.210; logERCC1: HR = 1.054, 95% CI = 0.852 - 1.304, *P* = 0.638) indicated that there was no significant association of DFS with the mRNA expression levels of *TS* and *ERCC1* in Chinese CRC patients treated with oxaliplatin and 5-FU based adjuvant chemotherapy (Table 2).

**Table 2:** **Univariate analyses of disease free survival according to the cox regression model**

Variable	Hazard ratio	95% confidence interval	***P***
logTS	0.820	0.602-1.118	0.210
logERCC1	1.052	0.851-1.302	0.638

logTS: the logarithms of the expression level of *TS*; logERCC1: the logarithms of the expression level of *ERCC1*.

Factors considered in the multivariate analyses included age, sex (male, female; reference category: female), tumor stage (stage IIa, stage IIb, stage IIIa, stage IIIb, stage IIIc; reference category: stage IIIc), tumor differentiation (high, high or medium, medium, medium or low, low; reference category: low), primary tumor location (rectum, colon; reference category: rectum), lymph node staging (N0, N1, N2; reference category: N2), vascular invasion (positive, negative; reference category: positive), nerve invasion (positive, negative; reference category: positive), interval between surgery and chemotherapy, duration of chemotherapy course, cumulated dosage of oxaliplatin as well as the mRNA expression levels of *TS* and *ERCC1.* It is clearly showed that only the tumor stage (tumor stage IIIc, reference, *P* = 0.083; tumor stage IIb, HR = 0.240, 95% CI = 0.080 - 0.724, *P* = 0.011; tumor stage IIc, HR < 0.0001, *P* = 0.977; Tumor stage IIIa, HR = 0.179, 95% CI = 0.012 - 2.593, *P* = 0.207) entered the model in the final step and was confirmed to be the independent prognostic factor for DFS. The results indicated that the DFS in the patients with tumor stage IIb was significantly longer than that in the patients with tumor stage IIIc. In addition, there was no evidence to prove the association between the DFS and the TS and ERCC1 mRNA levels (Table 3).

**Table 3:** **Cox regression analysis for multivariate analysis**

Variable	Disease free survival		
	Hazard ratio	95% confidence interval	***P***
Tumor stage (reference category: tumor stage IIIc)			0.083
Tumor stage IIb	0.240	0.080 - 0.724	0.011
Tumor stage IIc	< 0.0001	--	0.977
Tumor stage IIIa	0.179	0.012 - 2.593	0.207

For the tumor stage IIc, the hazard ratio was so small that the 95% confidence interval could not be displayed by the software.

**Figure 1:**
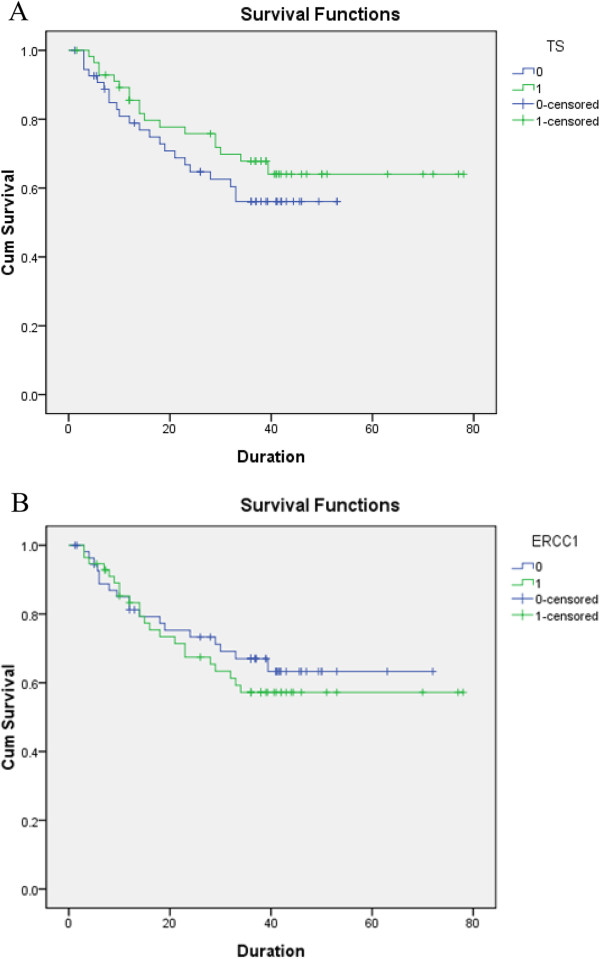
**Disease free survival curves according to the expression level of** ***TS*** **and** ***ERCC1*** **. A**: disease free survival curves according to the expression level of *TS*. **B**: Disease free survival curves according to the expression level of *ERCC1.* 0: low expression group; 1: high expression group.

The patients were divided into two groups based on the median mRNA expression levels of *TS* (high expression group: > 2.86 × 10^-1^; low expression group: ≤ 2.86 × 10^-1^) and *ERCC1* (high expression group: > 1.7 × 10^-3^; low expression group: ≤ 1.7 × 10^-3^). The Kaplan–Meier DFS curves according to the mRNA expression levels of *TS* and *ERCC1* all showed no significant difference between high and low expression group (*TS*: *P* = 0.264; *ERCC1*: *P =* 0.484), suggesting that the mRNA expression levels of *TS* and *ERCC1* was not significantly associated with the DFS (Figure 1).

## Discussion

The expression of *ERCC1* and *TS* has been reported to be related with the clinical outcomes of patients treated with the oxaliplatin or 5-FU-based adjuvant chemotherapy [24, 25]. Nevertheless, there was no enough evidence to prove the prognostic role of *ERCC1* and *TS* expression in CRC patients treated with oxaliplatin and 5-FU-based adjuvant chemotherapy. Therefore, we analyzed the association of mRNA expression levels of *ERCC1* and *TS* with DFS in Chinese patients with stage II-III CRC in this study. The results indicated no significant association between DFS and the mRNA expression levels of *ERCC1* and *TS*, suggesting that the expression of *ERCC1* and *TS* were not applicable as the predictive factors for DFS in Chinese stage II-III CRC patients receiving 5-FU and oxaliplatin based adjuvant chemotherapy.

However, the mRNA levels of *ERCC1* and *TS* has been reported by Shirota Y et al. to be associated with survival of 5-FU and oxaliplatin adjuvant chemotherapy in CRC patients [20]. It may be due to the difference in cancer stage and ethnicity of patients. In this study, the patients were at the stage II-III of CRC. However, the patients in the study of Shirota Y et al. [20] were at stage IV. The mRNA expression levels of *ERCC1* and *TS* may vary with different stage of cancer. In addition, the patients were all Chinese in this study but American in the study of Shirota Y et al. The gene expression profiles were different among ethnic groups [26]. Therefore, we inferred that the response of gene *ERCC1* and *TS* to oxaliplatin and 5-FU based adjuvant chemotherapy might be different between Chinese and American patients.

Some previous studies have reported that *ERCC1* expression is a predictive factor for survival after chemotherapy in advanced non-small cell lung cancer [27], bladder cancer [28], gastric cancer [24]. However, there was no evidence to prove the association between the mRNA expression of *ERCC1* and DFS of stage II-III CRC patients receiving oxaliplatin and 5-FU based adjuvant chemotherapy in this study. It indicated that *ERCC1* expression could predict clinical outcomes of chemotherapy in cancers such as non-small cell lung cancer, bladder cancer, and gastric cancer but not in stage II-III CRC.

Moreover, in this study, we found that tumor stage was a significant prognostic factor of DFS in CRC patients receiving 5-FU and oxaliplatin based adjuvant chemotherapy. It has been reported that the survival of advanced/recurrent rectal cancers treated with 5-FU based chemotherapy was significantly associated with the tumor stage [29]. Meanwhile, another studies reported that pathologic stage significantly influenced the DFS of locally advanced rectal cancer patients after preoperative chemoradiation (5-FU or oxaliplatin) [30]. Therefore, tumor stage must be considered in the further studies for the prognostic analysis of 5-FU and oxaliplatin based adjuvant chemotherapy for CRC patients.

There were some notable limitations of this study. First, power calculation (α = 0.05; *TS*: power 1 - β = 0.511; *ERCC1*: power 1 - β = 0.656) showed that the sample size was small for reliably accessing the association between *TS* or *ERCC1* expression and DFS. Thus, more studies with larger sample size were required. Second, the follow-up duration was short, so that further studies must be done to verify the results of this study. Third, there were four chemotherapy regimens in this study, which may be a limitation for identifying association between *TS* or *ERCC1* expression and DFS in this study. In addition, the dose of 5-FU may also affect the clinical outcomes of chemotherapy, and we must investigate this potentially prognostic factor in the further studies.

## Conclusions

In conclusion, our data demonstrated that mRNA expression levels of *ERCC1* and *TS* were not significantly correlated with the DFS of Chinese stage II-III CRC patients receiving 5-FU and oxaliplatin based adjuvant chemotherapy. It suggested that the mRNA expression levels of *ERCC1* and *TS* were not applicable as the predictive factors for DFS in Chinese stage II-III CRC patients receiving 5-FU and oxaliplatin based adjuvant chemotherapy. Further investigations to clearly define the role of *ERCC1* and *TS* gene expression in this setting are needed.
